# Elevated HIF-1α levels in maintenance hemodialysis patients: a potential link to increased cognitive impairment risk

**DOI:** 10.3389/fnagi.2024.1455596

**Published:** 2024-12-09

**Authors:** Lan Guo, Caiyun Jia, Ke Luo, Juanrong Liang, Lijun Wang, Tianli Hui

**Affiliations:** ^1^Department of Nephrology, Hebei General Hospital, Shijiazhuang, China; ^2^Department of Pharmacy, Hebei General Hospital, Shijiazhuang, China; ^3^Department of Nephrology, The First People’s Hospital of Tianmen City, Tianmen, China; ^4^Breast Center, The Fourth Hospital of Hebei Medical University, Shijiazhuang, China

**Keywords:** HIF-1α, maintenance hemodialysis, cognitive impairment, biomarker, risk

## Abstract

**Introduction:**

In China, an increasing number of patients with end-stage renal disease are undergoing hemodialysis treatment. While this treatment yields relatively positive outcomes, the prevalence of cognitive impairment in patients receiving maintenance hemodialysis ranges from 24 to 80%, which is significantly higher than the general population.

**Method:**

In this retrospective study, a total of 120 patients with kidney disease undergoing maintenance hemodialysis (MHD) were enrolled. The cognitive status of these patients was assessed using the C-MoCA score, which allowed categorization into two groups: the no cognitive impairment (NCI) group and the cognitive impairment (CI) group. Relevant clinical data, laboratory test results, as well as HIF-1α levels, were collected and analyzed to determine their relationship with the cognitive status of the patients.

**Results:**

In this study, a total of 45 patients (37.5%) developed CI, and their C-MoCA scores were significantly lower (21.6 ± 2.43) compared to patients in the NCI group (27.56 ± 1.48) (*P* < 0.001). The CI group was characterized by older age, lower levels of education, as well as lower levels of serum total bilirubin, serum total protein (TP), albumin, serum creatinine, and serum phosphorus in comparison to the NCI group. Additionally, CI patients exhibited higher levels of HIF-1α, received fewer monthly hemodiafiltration or hemoperfusion treatments, and had a lower rate of rosacastat treatment. Furthermore, univariate and multivariate logistic regression analyses demonstrated that older age (OR = 11.266 [95% CI: 2.775–45.747], *P* = 0.001) and higher HIF-1α (OR = 20.654 [4.831–88.298], *P* < 0.001) increased the risk of developing CI, while higher educational attainment reduced the risk of developing CI (> 12 years, OR = 0.004 [95% CI: 0.016–0.619], *P*≤0.001; 6–12 years, OR = 0.099 [95% CI: 0.000–0.049], *P* = 0.013).

**Discussion:**

Cognitive impairment in patients undergoing maintenance hemodialysis (MHD) was found to be associated with older age, lower level of education, and higher HIF-1α levels. These factors should be taken into consideration by clinicians to monitor the cognitive status of MHD patients.

## 1 Introduction

In recent years, the prevalence of diseases such as diabetes, obesity, and hypertension has been increasing, along with the aging population, leading to a rise in cases of end-stage renal disease (ESRD) every year (Shea et al., 2016). With the advancement of medical technology, the number of patients undergoing renal replacement therapy has significantly increased, and it is estimated that the number of people receiving renal replacement therapy will reach 5.439 million by 2030 (Iyasere et al., 2017). Currently, there are three available methods of renal replacement therapy–kidney transplantation, hemodialysis, and peritoneal dialysis–with the majority of ESRD patients in China opting for hemodialysis (Neuropsychology and Behavioral Neurology Group, Chinese Society of Neurology, Chinese Medical Association, 2019). Patients with chronic kidney disease (CKD) requiring dialysis have a much higher risk of cognitive impairment compared to the general population. The incidence of cognitive impairment among those undergoing maintenance hemodialysis (MHD) ranges from approximately 24 to 80%.

Patients suffering from cognitive impairment (CI) concurrent with MHD might progressively experience a decline in cognitive functions, including attention, memory, and executive skills (Drew et al., 2017). Such cognitive deficiencies can diminish the patient’s compliance with treatment, influencing their capacity to self-manage and the control of complications, thereby eventually affecting their survival rate and quality of life (Orock et al., 2020). Dialysis patients with CI require more time for dialysis, extended hospital stays, and face a higher mortality risk. A study conducted in Italy (Matsubara et al., 2017) revealed that among dialysis patients without CI, the mortality rate was 5.1 deaths per 100 person-years, while for those with CI, the mortality rate was 12.6 deaths per 100 person-years (HR = 2.49). Early diagnosis, identification of risk factors affecting cognitive impairment, and timely implementation of intervention measures can help improve the patient’s cognitive function status.

The diagnosis of CI may be identified based on patients’ medical history, neuropsychological assessments, neurophysiology, and images for CI diagnose. However, these methods are time-consuming and costly, and require a high level of expertise from clinicians (Wang et al., 2018). Currently, high-sensitivity neuropsychological tests can be used to screen patients for mild cognitive impairment (MCI), thereby facilitating early detection of cognitive abnormalities. In clinical practice, the Chinese version of the Montreal Cognitive Assessment (C-MoCA) and the Mini-Mental State Examination (C-MMSE) are frequently used in clinical practice to assess overall cognitive function. Patients who do not attend related neuropsychological outpatient clinics may be overlooked in CI diagnosis. Hence, the development of diagnostic biomarkers could potentially increase early detection rates of CI.

HIF-1α, with a molecular weight of 120 kd, cannot be effectively removed through standard dialysis methods. When exposed to hypoxic conditions, HIF-1α plays a crucial role in regulating energy metabolism, counteracting oxidative stress, promoting the growth of new blood vessels, and facilitating cellular adaptation to low oxygen levels (He et al., 2017). Consequently, under hypoxic conditions, HIF-1α may enhance cerebral perfusion. A study on thoracic surgery revealed that longer durations of one-lung ventilation resulted in higher serum levels of HIF-1α. Furthermore, an increased level of HIF-1α was associated with a higher incidence of postoperative cognitive dysfunction, some researchers suggested that early expression of HIF-1α may provide a protective effect against the onset of cognitive dysfunction (Kurella Tamura et al., 2013). Additionally, patients with obstructive sleep apnea-hypopnea syndrome (OSAHS) who exhibited higher levels of HIF-1α, showed greater degree of airway blockage and the severity of decision-making dysfunction (Ozcan et al., 2015). In patients with traumatic brain injury (TBI), those who underwent hyperbaric oxygen therapy (HBOT) exhibited decreased levels of HIF1α alongside improved cognitive function scores (Liu et al., 2023). Furthermore, in Alzheimer’s disease (AD) models, increased expression of HIF1α correlates with a decline in cognitive function (Zhang et al., 2020). Additionally, intermittent hypoxia (IH), a characteristic manifestation of obstructive sleep apnea (OSA), leads to increased expression of HIF-1α, which is associated with the development of hypertension, type 2 diabetes, and cognitive decline (Prabhakar et al., 2020).

Based on these findings, this research aimed to examine clinical variables associated with CI and investigate the relationship between HIF-1α and CI in patients undergoing maintenance hemodialysis (MHD). Furthermore, this study aims to explore the potential of HIF-1α as a molecular biomarker for predicting CI.

## 2 Materials and methods

### 2.1 Patient enrollment

This study recruited 120 patients who were treated in the Department of Nephrology, Hebei Provincial People’s Hospital, from August to November, 2021. The inclusion criteria were as follows: MHD patients aged ≥ 18 years; Underwent regular maintenance hemodialysis (MHD) for a minimum of three months; Finished HIF-1α test; Clinically stable and completed cognitive assessments; Stored serum samples available. Exclusion criteria were: Congenital intellectual disabilities; History of mental illness or severe complications leading to significant cognitive impairment; The use of medications for mental disorders that could impact cognitive function; Incomplete test data. This study was approved by the Ethics Committee of Hebei Provincial People’s Hospital (2022158).

### 2.2 Data collection

The venous blood was collected in the morning on an empty stomach prior to dialysis. The blood samples were then analyzed using a fully automated biochemical analyzer to detect various factors. These included white blood cells (WBC), red blood cells (RBC), platelet (PLT), hemoglobin (Hgb), total protein (TP), albumin (Alb), glucose (GLU), serum creatinine (Scr), blood urea nitrogen (BUN), total bilirubin (Tbil), uric acid (UA), intact parathyroid hormone (iPTH), serum calcium (Ca), serum phosphorus (P), β2-microglobulin (β2-MG), Interleukin-6 (IL-6), C-reactive protein (CRP), serum iron (Fe), serum ferritin (SF).

### 2.3 HIF-1α analysis

Peripheral blood was collected from patients in a fasting state prior to dialysis. The levels of HIF-1α in the patients’ blood were analyzed using the Enzyme-Linked Immunosorbent Assay (ELISA), following the instructions of a commercial HIF-1α test kit (Heppon Shanghai Biotechnology Co., Ltd., Cat.202110). Concentrations of HIF-1α were determined by detecting signals using an ELISA reader (Labsystems, MUTISKAN). The patients were divided into two groups based on the abundance of HIF-1α: low-HIF-1α group and high-HIF-1α abundance group. The median level of HIF-1α was used as the cut-off value for this categorization.

### 2.4 Cognitive function evaluation

The Chinese version of the Montreal Cognitive Assessment scale (C-MoCA) was used to assess cognitive function. To reduce the influence of subjective factors and minimize assessment errors caused by language and comprehension issues, a single physician, who had received standardized training in using C-MoCA, performed the evaluation within one hour prior to the MHD procedure. In order to avoid bias arising from different educational levels, an additional point was added to the C-MoCA score for participants with less than 12 years of education. According to the C-MoCA scoring results, patients with total scores of < 26 points were classified as having cognitive impairment (CI), and those with scores ≥ 26 points were categorized within the cognitively normal group (Drew et al., 2015).

### 2.5 Statistical analysis

Firstly, data were tested for normality: normally distributed quantitative data were reported as mean ± standard deviation, and differences between groups were compared using independent sample *t*-tests or non-parametric rank sum tests. Non-normally distributed quantitative data were compared between groups using a non-parametric rank sum test. Data that showed significant differences between groups were further analyzed using the Spearman rank correlation and logistic regression analyses. A *P*-value < 0.05 was considered statistically significant. The software of SPSS24.0 was used for statistical analysis.

## 3 Results

### 3.1 Patient characteristics

**FIGURE 1:**
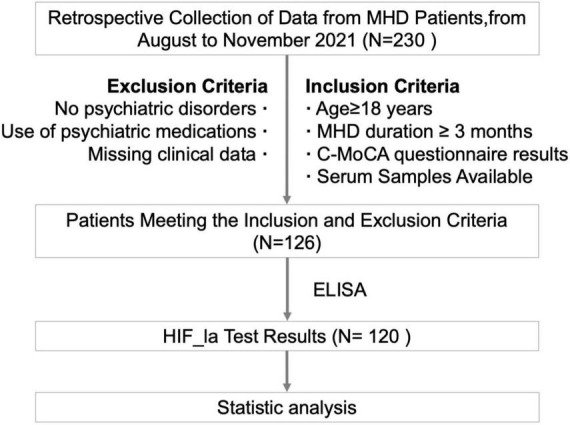
The workflow of the study.

The workflow of patient enrollment was shown in Figure 1. 120 patients were included in this study and the baseline characteristics are shown in Table 1. The median age was 60 years (range: 32–84 years), with 73 male (60.8%) and 47 female (39.2%) patients. In terms of education level, 20 (16.7%) had less than 6 years of education, 62 (51.7%) had 6–12 years, and 38 (32%) had more than 12 years. The median duration of dialysis was 25.5 months (range: 3–155 months). The primary causes of kidney failure were as follows: primary glomerulonephritis in 52 (43.3%) patients, diabetic nephropathy in 35 (29.1%), polycystic kidney disease in 10 (8.3%), ANCA-associated vasculitis in 5 (4.2%), nephrotic syndrome in 5 (4.2%), IgA nephropathy in 3 (2.5%), hypertensive renal damage in 3 (2.5%), anti-GBM disease in 3 (2.5%), lupus nephritis in 2 (1.7%), and obstructive nephropathy in 2 patients (1.7%).

**TABLE 1:** Characteristics and group differences between patients with (CI group) and without cognitive impairment (NCI group).

Variables		NCI group (*n* = 75)	CI group (*n* = 45)	*P*
Age (year)		51.87 ± 13.18	65.02 ± 8.99	< 0.001*
Gender	Male	50 (66.67%)	23 (51.11%)	0.092^#^
	Female	25 (33.33%)	22 (48.89%)	
Education level	< 6 years	3 (4%)	17 (37.78%)	< 0.001^#^
	6–12 years	36 (48%)	26 (57.78%)	
	> 12 years	36 (48%)	2 (4.44%)	
Stroke	Yes	21 (28%)	20 (44.44%)	0.067^#^
	No	54 (72%)	25 (55.56%)	
Take roxadustat *N* (%)	Yes	22 (29.33%)	5 (11.11%)	0.021^#^
	No	53 (70.67%)	40 (88.89%)	
Hemodiafiltration	Twice/month	23 (30.67%)	15 (33.33%)	< 0.001^#^
	Once/month	24 (32%)	15 (33.33%)	
	No	28 (37.33%)	15 (33.33%)	
Hemoperfusion	Twice/month	15 (20%)	2 (4.44%)	0.016^#^
	Once/month	9 (12%)	4 (8.89%)	
	No	51 (68%)	39 (86.67%)	
Hemoglobin (g⋅L^–1^)		108.8 ± 19.57	112.62 ± 17.1	0.28
Total protein (g⋅L^–1^)		74.87 ± 5.85	69.45 ± 5.89	< 0.001*
Albumin (g⋅L^–1^)		41.37 ± 2.93	38.45 ± 4.15	< 0.001*
Total bilirubin (μmol⋅L^–1^)		11.89 ± 4.21	10.38 ± 2.71	0.034*
Serum creatinine (μmol⋅L^–1^)		939.05 ± 285.08	755.94 ± 197.59	< 0.001*
Serum phosphorus (mmol⋅L^–1^)		2.12 ± 0.65	1.87 ± 0.58	0.037*
HIF-1α (pg⋅ml^–1^)		1,272.26 ± 213.66	1,532.76 ± 211.44	< 0.001*
Dialysis duration (month)		19 (11, 62)	30 (13, 60)	0.024^&^
Intact parathyroid hormone		239.8 (147.9, 365.6)	261 (179.85, 365.25)	0.653^&^
Serum iron (mmol⋅L^–1^)		11.1 (9.23, 15.65)	10.40 (7.6, 15.6)	0.494^&^
Serum ferritin (mmol⋅L^–1^)		293.55 (171.23, 429.7)	324.9 (168.1, 511.15)	0.653^&^

*t-test was used for comparing the means.

^#^Chi-square test was used to compare categorical variables.

^&^Non-parametric rank sum test to compare medians between two independent samples.

### 3.2 Comparison of C-MoCA scores between CI and NCI groups

Within the cohort of 120 patients undergoing maintenance hemodialysis (MHD), a total of 75 individuals (62.5%) exhibited normal cognitive function, while 45 individuals (37.5%) displayed cognitive impairment. The mean C-MoCA score in the cognitive impairment group (27.56 ± 1.48) was significantly higher than in those with cognitive impairment (21.6 ± 2.43) (*P* < 0.001).

**FIGURE 2:**
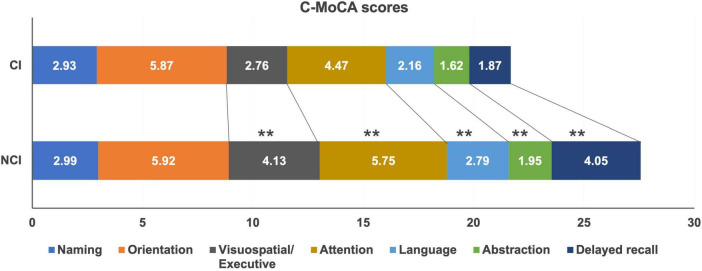
Comparison of C-MoCA scores between CI and NCI groups. **P < 0.01.

To comprehensively evaluate different aspects of cognitive impairments among the patients, the C-MoCA scoring tool was employed, and the findings are presented in Figure 2. The scores for visuospatial and executive function (*P* < 0.001), attention (*P* < 0.001), language (*P* < 0.001), abstraction (*P* < 0.001), and delayed memory (*P* < 0.001) exhibited significant reductions in the cognitive impairment (CI) group compared to those with normal cognitive function. However, there were no statistically significant differences in the scores for naming and orientation between the two groups (*P* > 0.05).

### 3.3 The distribution of factors between CI and NCI groups

To compare the characteristics of patients with normal cognitive function and those with cognitive impairment, various patient data such as age, gender, education level, medical history, treatment, and laboratory test results were collected and analyzed for distribution differences between the two groups. The findings are presented in Table 1. Compared to the NCI group, patients in the CI group displayed several distinguishing features. Firstly, they were older in age, with a mean age of 65 compared to 52 in the NCI group (*P* < 0.001). Additionally, the CI group had higher levels of serum HIF-1α (1,532.76 vs. 1,272.26, *P* < 0.001), lower levels of total protein (TP) (69.45 vs. 74.87, *P* < 0.001), lower levels of albumin (A1b) (38.45 vs. 41.37, *P* < 0.001), lower levels of total bilirubin (Tbil) (10.38 vs. 11.89, *P* = 0.034), and lower levels of blood creatinine (Scr) (755.94 vs. 939.05, *P* < 0.001). Furthermore, a majority of patients in the CI group (86.67%) did not receive hemoperfusion treatment, which was statistically significant (*P* = 0.016).

In terms of education level, there was a significant difference between the two groups (*P* < 0.001). Only a small number of patients in the CI group (*N* = 2, 4.44%) had an education duration of more than 12 years, while a majority of patients in the NCI group (48%) had received education for more than 12 years. Additionally, more patients in the NCI group had taken roxadustat compared to the CI group (29 vs. 11%, *P* = 0.021). However, there were no statistically significant differences between the two groups in terms of gender, and history of stroke (*P* > 0.05).

### 3.4 Correlation analysis of factors affecting cognitive function in MHD patients

To further explore the clinical factors associated with cognitive impairment, we performed a correlation analysis between cognitive function and the factors that exhibited significant distribution differences in the two groups. The findings indicated a statistically significant association between these factors and CI (Table 2). Specifically, TP (*r* = −0.412) and education level (*r* = −0.584) exhibited a negative correlation with CI, suggesting that higher TP levels and education levels were linked to a lower likelihood of CI occurrence. Conversely, age (*r* = 0.467) and HIF-1 (*r* = 0.509) demonstrated a positive correlation with CI, implying that older age and higher HIF-1 levels were associated with a higher likelihood of CI occurrence.

**TABLE 2:** Analysis of the correlation between factors and CI.

Variables	*r*	*P*
*P* (mmol⋅L^–1^)	−0.186	0.042
Tbil (μmol⋅L^–1^)	−0.195	0.032
Scr (μmol⋅L^–1^)	−0.297	0.001
TP (g⋅L^–1^)	−0.412	< 0.001
Alb (g⋅L^–1^)	−0.357	< 0.001
Age (year)	0.467	< 0.001
HIF-1α (pg⋅ml^–1^)	0.509	< 0.001
Roxadustat	−0.205	0.025
Hemodiafiltration times	−0.334	< 0.001
Hemoperfusion times	−0.220	0.016
Education level	−0.584	< 0.001

The meaning of each abbreviation is detailed in the English abbreviation table above. The spearman correlation was shown in continuous variables, and Kendall test correlation were shown in rank variables.

### 3.5 Logistic regression analysis of factors affecting cognitive function in MHD patients

**FIGURE 3:**
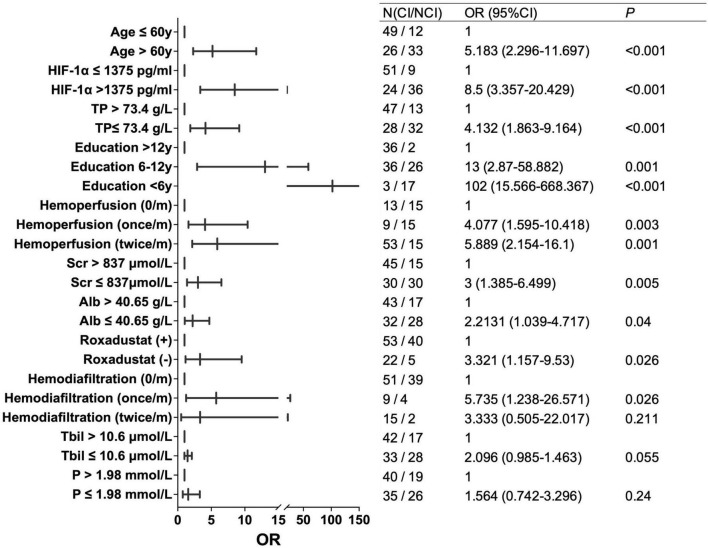
The OR forest plot of risk factors that influenced cognitive function in MHD patients.

For further analysis, variables with a correlation coefficient | r| > 0.4, including TP, Age, HIF-1α, and education level, were selected for univariate logistic regression analysis. Patients were divided into two groups based on the median values of TP, A, ge, and HIF-1α, respectively. The results of the univariate logistic regression analysis showed that patients in the high HIF-1α group (OR = 8.5, *P* < 0.001), and older patients (> 60) (OR = 5.183, *P* < 0.001) had higher risks of CI. Conversely, a higher education level (> 12 years) was associated with a decreased risk of CI (*P* < 0.001), and a higher TP level was associated with a reduced risk of CI (OR = 4.132 for TP ≤ 73.4 g/L, *P* < 0.001) (Figure 3).

Multiple logistic regression analysis was conducted on HIF-1α, age, education level, and TP, these variables achieved a *P*-value of less than 0.001 in univariate analysis. As a result, age, HIF-1α, and education level were found to be independent risk factors for cognitive impairment. However, TP did not exhibit a statistically significant influence on cognitive impairment (Table 3).

**TABLE 3:** Multivariate logistic regression analysis of cognitive function in MHD patients.

		B	SE	OR	95% CI	*P*
HIF-1α (pg⋅ml^–1^)	Low			1		< 0.001
	High	3.028	0.741	20.654	4.831–88.298	
Age (year)	< 60			1		0.001
	> 60	2.422	0.715	11.266	2.775–45.747	
Education level	< 6 years			1		
	6–12 years	−2.309	0.934	0.099	0.016–0.619	0.013
	> 12 years	−5.578	1.312	0.004	0.000–0.049	< 0.001
TP (g⋅L^–1^)	Low			1		0.096
	High	−1.024	0.615	0.359	0.108–1.20	

## 4 Discussion

Previous research has demonstrated that the incidence of cognitive impairment among patients undergoing maintenance hemodialysis (MHD) ranges from 24 to 80% (Griva et al., 2006; Harciarek et al., 2009; Drew et al., 2015; Sarnak et al., 2013). Consistent with these findings, our study revealed a 37.5% incidence rate of cognitive impairment among a sample of 120 MHD patients. The aim of this study was to investigate factors contributing to the development of cognitive impairment in MHD patients. Therefore, we retrospectively collected patient data from our hospital, including clinical demographics, blood and urine analyses, and other laboratory parameters. Our findings indicate that advanced age, lower educational attainment, and elevated HIF-1α levels are correlated with an increased risk of cognitive impairment.

Between the CI group and NCI group, statistically significant differences (*P* < 0.05) were observed in the areas of visuospatial and executive function, attention, language, abstract, and delayed memory. Notably, during the administration of cognitive tests, we noticed that nearly all MHD patients performed well on evaluations of orientation, this may due to their regular weekly visits to the hospital for dialysis. However, patients showed variance in areas of execution and attention. Consequently, we speculate that repeated exposure to stimuli associated with orientation, along with tailored reinforcement training, may have the potential to prevent cognitive impairment in MHD patients.

Previous researches of age-related neurodegenerative changes have found that age is a significant risk factor affecting cognitive decline (Ogunshola and Antoniou, 2009; Correia and Moreira, 2010; Wardlaw et al., 2013). The occurrence rate of cognitive impairment significantly increases among the patients aged between 65 and 79 years (Ogunshola and Antoniou, 2009). As age increases, the occurrence of principal diseases, including cardio-cerebrovascular complications, brain atrophy, and brain injuries, raises the risk of cognitive damage. Our study also supports the finding that older patients face a higher risk of developing cognitive impairment. Therefore, it is crucial to closely monitor changes in cognitive function among elderly patients who need dialysis. In this study, the median age of the patients included was 60 years (range, 32 to 84). For patients younger than 60 years, those with elevated HIF1a levels had a risk of CI of 7.895 (*P* = 0.013). For patients aged 60 years and above, the risk of CI in the group with high levels of HIF1a was 15.6 (*P* < 0.001). This suggests that increased HIF1a levels are associated with an increased risk of CI in both younger and older patient groups.

Furthermore, our study revealed that the CI group had lower levels of total protein and albumin compared to the NCI group. Albumin levels reflect the patient’s nutritional status, and its decline can decrease plasma colloid osmotic pressure. Consequently, there is capillary dilation and a large amount of fluid infiltrates the tissue interstitial space from the blood vessels, resulting in tissue interstitial edema and serous cavity edema. This condition reduces the effective circulating blood volume and increases vascular resistance, ultimately leading to decreased perfusion of vital organs such as the brain and heart, thereby exacerbating the progression of cognitive impairment (Ogle et al., 2012). While some observational studies suggest a link between elevated serum parathyroid hormone (PTH) levels and an increased risk of cognitive impairment or dementia, in this study, we did not observe differences in PTH levels between the CI and NCI groups. The relationship between PTH and CI remains inconclusive, a large population-based cohort study that found no independent influence of PTH on cognitive decline over a 20-year period (Kim et al., 2017). In this study, we did not conduct a more in-depth analysis of the relationship between PTH and CI.

Previous researches have indicated that hemoglobin play a crucial role in cognitive impairment (Wolfgram et al., 2015; Murayama et al., 2017; Siebert et al., 2018). According to the World Health Organization (WHO) criteria, anemia is defined as a hemoglobin concentration of less than 130 g/L in men and less than 120 g/L in women (World Health Organization, 1994). In our study, the mean hemoglobin levels in both the CI and NCI groups were below 120 g/L. However, no statistically significant difference in hemoglobin levels was observed between the two groups. Some patients were administered Roxadustat, a hypoxia-inducible factor prolyl hydroxylase inhibitor (HIF-PHI), which functions by inhibiting the degradation of HIF-1α, thereby increasing its levels and ameliorating anemia. Notably, a higher proportion of patients with normal cognition were found had taken Roxadustat, with this difference reaching statistical significance (*P* < 0.05). However, only 27 patients in our study were treated with Roxadustat, which constitutes a relatively small sample size. Therefore, further research is needed to validate the relationship between rosuvastatin and cognitive impairment in MHD patients. Patients undergoing MHD are chronically exposed to mild inflammation and chronic ischemic hypoxia, both of which can cause bodily harm while activating endogenous adaptive compensatory responses. We hypothesize that HIF-1α may act as a protective factor, increasing compensatory secretion to counteract the adverse effects of hypoxia and mild inflammation, thereby exerting a protective role. Under hypoxic conditions, HIF-1α binds to targeted downstream genes, regulating energy metabolism, combating oxidative stress, promoting angiogenesis, and facilitating cellular adaptation to hypoxic environments (He et al., 2017). Consequently, increased HIF-1α under hypoxic conditions could enhance brain perfusion and aid cells in adapting to hypoxic stress. Although this study did not measure the oxygen levels in the patients’ blood, the mean hemoglobin content in MHD patients is lower than that in healthy individuals, potentially leading to hypoxia within the patient’s body.

In this study, the CI group exhibited higher levels of HIF-1α compared to the NCI group. To determine whether this difference is related to variations in oxygen levels, we analyzed the hemoglobin, serum iron, and serum ferritin levels between the CI and NCI groups, but no significant difference was found. Given the absence of direct measurements of oxygen saturation in this study, we cannot conclusively determine the relationship between systemic hypoxia and elevated HIF-1α levels at this time. Furthermore, the relationship between elevated HIF-1α levels and neuroprotection remains unclear. The role of HIF-1α may vary depending on the degree and stage of ischemia, and a consensus on this issue has yet to be reached. Van Zwieten et al. (2019) demonstrated that the upregulation of HIF-1α can enhance the expression of vascular endothelial growth factor (VEGF) and erythropoietin (EPO), which subsequently leads to increased local cerebral blood flow and a reduction in the extent of cerebral infarction following ischemic stroke in animal models. Our current study does not clarify whether the elevation of HIF-1α is a cause of CI or a consequence of CI. Future longitudinal cohort designs, with prospective collection of more comprehensive clinical data affecting HIF-1α and CI, may provide evidence for the relationship between HIF-1α and CI.

There are several limitations in this study. Firstly, some patients were unable to undergo cognitive assessments due to visual and hearing impairments. Additionally, some patients were aware of their cognitive impairment and may have had psychological resistance to the testing, which could have influenced their willingness to participate in cognitive function assessments. Secondly, our study design was cross-sectional, limiting our ability to observe changes in cognitive function over time as relevant indicators fluctuate. Lastly, due to the retrospective nature of our study, we were unable to include certain factors known to affect cognitive function, such as vitamin D deficiency, thyroid dysfunction, and hyperhomocysteinemia, due to the lack of available data.

In conclusion, the prevalence of cognitive impairment in patients undergoing MHD is notably high, and it is important to monitoring of cognitive status in these individuals. Our findings suggest a positive correlation between HIF-1α levels and the incidence of cognitive impairment. HIF-1α is an independent risk factor for cognitive impairment. Additionally, for elderly patients or those with low levels of education who undergo MHD are at a higher risk of CI. Clinicians may pay more attention on individuals undergoing MHD with higher levels of hypoxia-inducible factor-1 alpha (HIF-1α), advanced age, lower level of education for early detection and intervention of CI.

## Data Availability

The raw data supporting the conclusions of this article will be made available by the authors, without undue reservation.
